# Clinical and Radiographic Manifestations of Sputum Culture-Negative Pulmonary Tuberculosis

**DOI:** 10.1371/journal.pone.0140003

**Published:** 2015-10-08

**Authors:** Minh-Vu H. Nguyen, Elizabeth R. Jenny-Avital, Susanne Burger, Eric M. Leibert, Jacqueline M. Achkar

**Affiliations:** 1 Division of Infectious Diseases, Department of Medicine, Albert Einstein College of Medicine, Bronx, New York, United States of America; 2 Division of Pulmonary and Critical Care Medicine, Department of Medicine, New York University School of Medicine, New York, New York, United States of America; 3 Department of Medicine, Albert Einstein College of Medicine, Bronx, New York, United States of America; 4 Department of Microbiology and Immunology, Albert Einstein College of Medicine, Bronx, New York, United States of America; Public Health Research Institute at RBHS, UNITED STATES

## Abstract

Intervention at the earliest possible stage of pulmonary tuberculosis (PTB) reduces morbidity for the individual and transmission for the community. We characterize the clinical and radiographic manifestations of sputum culture-negative (Cx-) PTB in order to facilitate awareness of this under recognized and likely early disease state. In this cross-sectional sub-study, we reviewed the medical records of HIV-uninfected PTB patients enrolled from 2006–2014 within the context of a TB biomarker study in New York City. Cx- PTB was defined as clinical and/or radiographic presentation consistent with PTB, three initial mycobacterial sputum cultures negative, and no evidence of other respiratory disease. Diagnosis was confirmed by clinical and radiographic improvement on antituberculous treatment and/or culture, nucleic acid, or histological confirmation from a specimen other than the initial three sputa. Cx+ PTB was defined as above but with *M*. *tuberculosis* growth in at least one of the first three sputum cultures. Demographics, symptoms, and radiographic findings on initial presentation were compared between the two groups. Of 99 subjects diagnosed with PTB, 21 met the criteria of Cx- PTB. Cx- compared to Cx+ subjects presented with a significantly lower frequency of cough (70% vs. 91%, *P* = 0.02), sputum production (30% vs. 64%, *P* < 0.01), weight loss (25% vs. 54%, *P* = 0.02), and frequency of cavitation on chest CT (12% vs. 68%, *P* < 0.01). Our findings should raise awareness that neither a positive culture nor the hallmark symptoms are invariably associated with early TB disease.

## Introduction

With an estimated nine million new cases and about 1.5 million associated deaths annually, active tuberculosis (TB) continues to be a major cause of morbidity and mortality worldwide [1]. Early diagnosis with prompt treatment is critical for the reduction of *Mycobacterium tuberculosis* (Mtb) transmission, and thus is cornerstone to TB control. The gold standard for TB diagnosis continues to be confirmation by culture or nucleic acid amplification assay, with culture typically being the more sensitive method [2, 3]. However, in settings with adequate resources allowing for comprehensive diagnostic work-up and the exclusion of other respiratory diseases, a substantial proportion of TB cases is reported to be culture-negative (Cx-). The Centers for Disease Control and Prevention (CDC) reports that around 23% of TB cases in the United States (US) were Cx- in 2013 [4], and suggests that such cases may be due to “low bacillary populations” [5].

Cx- TB is likely to be less transmissible and more amenable to treatment with fewer drugs for shorter treatment duration. Thus, a low threshold for starting empiric antituberculous therapy is encouraged in patients with a high suspicion for Cx- TB and no evidence of other respiratory diseases. Dutt et al. showed that the relapse rate for an abbreviated four month treatment for Cx- TB was the same as that of standard 12 month therapy for Cx+ TB [6]. In agreement with these data, the American Thoracic Society and CDC state that the treatment for Cx- TB can be shortened from six to four months by reducing the continuation phase of isoniazid and rifampin from four to two months [5]. The benefit of preventing progression to advanced contagious TB, and the greater ease of treating Cx- TB, make the identification of this antecedent disease state an important priority. Despite the estimated considerable incidence of Cx- TB, little is known about its clinical manifestations. An early 1980s Hong Kong based study found that Cx- compared to smear-negative (Sm-) culture-positive (Cx+) pulmonary TB (PTB) was associated with less frequency of hemoptysis and radiologic abnormalities [7], but other studies validating such data are lacking.

Radiographically demonstrable disease in asymptomatic Cx- HIV-uninfected individuals can be construed as an early disease state based on its likelihood of progression despite a low mycobacterial burden (reviewed in [8]). In a control arm of a study of chemotherapy for radiographically typical but Sm- Cx- PTB in Hong Kong, progression ensued in 93/176 (53%) with culture confirmation in 70/93 (75%) subjects [7]. Similarly, a 1985 prospective study of untreated South African miners undergoing regular chest X-rays demonstrated that individuals with new or worsening radiographic abnormalities had a high likelihood of progression to Cx+ disease (58%) over three to 58 months with none developing a non-TB disease [9].

Absent a gold standard, studies characterizing Cx- PTB are best performed in settings where an exhaustive radiological, microbiologic, pathological and serologic evaluation can be undertaken leading to a definitive diagnosis other than TB, or to a TB diagnosis based on specimens other than the initial three sputum samples. In New York City (NYC), a locale with both a high burden of TB in its large foreign born population and ample resources for an exhaustive diagnostic evaluation, approximately 27% of TB cases are reported to be Cx- [10].

We hypothesized that Cx- PTB patients present with fewer symptoms, shorter symptom duration, and less radiographic abnormalities compared to those with Cx+ PTB. Therefore, our primary objectives were (1) to assess the presence and duration of TB-associated symptoms in Cx- compared to Cx+ PTB patients; and (2) to compare the radiographic abnormalities of Cx- to those of Cx+ PTB. Our secondary objectives were (1) to assess whether there are differences in demographic characteristics between Cx- and Cx+ PTB patients; and (2) to investigate trends in clinical and radiographic characteristics when comparing Cx-, Sm- Cx+, and smear-positive (Sm+) Cx+ PTB in a three-group analysis, with specific comparisons of each smear group with Cx- TB.

## Methods

### Study design, setting, and population

In this cross-sectional sub-study, we reviewed the medical records of patients diagnosed with PTB during the time of July 2006 through July 2014 in four public hospital centers in NYC (Bellevue Hospital Center, Jacobi Medical Center, Montefiore Medical Center, and North Central Bronx Hospital). The subjects were all TB suspects who were consecutively enrolled within the context of an ongoing TB biomarker study [11]. Inclusion criteria for our study were age ≥ 21 years, diagnostic work-up of three sputum samples with microscopy of sputum smears for acid-fast bacilli (AFB) and mycobacterial cultures. Exclusion criteria were HIV co-infection, current antituberculous treatment for > 2 weeks or a history of such TB treatment within a year prior to enrollment, and extrapulmonary disease without chest X-ray findings consistent with PTB.

This study was conducted in accordance with the amended Declaration of Helsinki, approved by the institutional review boards of the Albert Einstein College of Medicine and the New York University School of Medicine. Written informed consent was obtained from all patients.

### Measurements, diagnoses, and definitions

Following the guidelines provided by American Thoracic Society and the CDC [2, 12], and those by the World Health Organization (WHO) [13], subjects were considered PTB cases if they had “clinical, bacteriological, and/or radiographic evidence of current tuberculosis” in the lungs, and had been treated by a full course of antituberculous treatment. A diagnosis of Cx- PTB further required no growth of any mycobacteria in the first three sputum cultures as per CDC criteria [5], upper-lobe and/or miliary infiltrates on either chest X-ray or CT, and extensive work-up that excluded other respiratory diseases. In addition to chest CT in most subjects, extensive work up included tests such as aerobic and anaerobic sputum cultures for bacterial, parasitic and fungal organisms, *Legionella*, *Histoplasma*, and pneumococcal urine antigen tests as they became FDA approved and available, and serology for fungal and parasitic organisms as indicated such as serum antigens for *Cryptococcus neoformans* or serum antibodies for *Strongyloides*. Cx- PTB was confirmed by an Mtb-positive culture, nucleic acid detection, or histological diagnosis in a specimen other than the initial three sputa, or, as per CDC recommendation, clinical and/or radiographic improvement attributable to antituberculous treatment [5]. Diagnosis of Cx+ PTB was based on Mtb growth in at least one of the first three sputum cultures. Among the Cx+ subjects, Sm- PTB was defined as no AFB identified in the initial three sputum smears, while Sm+ PTB was defined as at least one positive AFB smear (regardless of quantity). As standard care in NYC, cough was induced by nebulized hypertonic saline to collect adequate sputum.

Demographic measurements included age, sex, race, foreign-birth, and time in the US among foreign-born subjects. Symptoms included subject-reported presence and duration of cough, sputum production, fever, night sweats, weight loss > 1.81 kg, and hemoptysis at the time of initial presentation. Radiological findings were based on the first chest X-ray obtained at initial presentation, and chest computer tomography (CT) scan if performed upon initial presentation. The location, extent and characteristics (nodular, miliary, cavitary) of radiographic pulmonary abnormalities were recorded.

### Sputum microscopy and mycobacterial cultures

Sputa were processed in the local hospital laboratories as per CDC guidelines [2]. Mycobacterial growth and species were identified using the BACTEC 460 or 960 MGIT detection systems (Becton Dickinson Microbiology Systems, Sparks, MD).

### Statistical analysis

Statistical analysis was performed using STATA software, version 13.1 IC (StataCorp, College Station, TX). The α was set at < 0.05. Normality and homoscedasticity assumptions were assessed for continuous variables, which were analyzed by the *t* test or Mann-Whitney *U* test for two-group and the ANOVA or Kruskal-Wallis rank test for three-group comparisons. Categorical variables were compared by the Pearson’s chi-square test without correction for continuity or the Fisher’s exact test.

## Results

Of 113 subjects diagnosed with TB, 14 extrapulmonary-only cases were excluded. Of the 99 subjects included for analysis, 21 had Cx- PTB. All Cx- PTB patients had at least one major TB risk factor (history of a positive tuberculin skin test or origin from a TB-endemic country) and had upper-lobe and/or miliary infiltrates on either chest x-ray and/or CT. Of these 21, 14 had Mtb confirmed in another specimen other than the initial three sputa by either culture, nucleic acid techniques, or histology consistent with TB (Table 1). Among the 78 Cx+ subjects, 25 were Sm- and 53 were Sm+, of which three had no AFB on their initial two smears, and were only categorized as Sm+ based on their third positive smear.

**Table 1:** Confirmation of Tuberculosis among Sputum Culture-Negative Cases (n = 21).

Confirmation Method		n (%)
Positive Mtb culture in a specimen other than the three initial sputum samples		9 (43)
	Bronchoalveolar lavage fluid	4
	Fourth sputum sample or beyond	2
	Lymph node biopsy^a^	2
	Pleural fluid	1
Mtb nucleic acid detection in a specimen other than the three initial sputum samples		3 (14)
	Fourth sputum sample or beyond	2
	Lung biopsy	1
Histological confirmation via lung biopsy^b^		2 (10)
Clinical and radiographic manifestations improved on antituberculous treatment		7 (33)

All sputum culture-negative cases had upper-lobe and/or miliary infiltrates on either chest x-ray and/or CT in addition to the above findings.

^a^ One neck, one mediastinal lymph node.

^b^ Caseating granulomas with acid-fast bacilli.

### Demographics and Symptomatology

No significant demographic differences were observed between Cx- and Cx+ subjects (Table 2). Ten subjects lacked information on symptoms, one was Cx-. Cx- subjects had a significantly lower frequency of cough, sputum production, weight loss, and any symptom in general compared to Cx+ subjects (p < 0.05; Table 2). The median duration of symptoms for Cx- subjects was 4 weeks and that of Cx+ subjects was 6 weeks, but the difference was not statistically significant. In the three-group comparison, there were significant differences in frequency of cough, sputum production, weight loss, and any symptom (Table 2), mostly driven by the differences between Cx- compared to Sm+ Cx+ subjects. For most symptoms, a trend for increasing frequency from Cx- to Sm- Cx+ to Sm+ Cx+ PTB was observed, with significant trends in cough (*P* for trend = 0.01), sputum production (*P* for trend < 0.01), weight loss (*P* for trend = 0.01), and any symptom (*P* for trend = 0.01).

**Table 2:** Demographics and Symptoms of Patients with Pulmonary Tuberculosis.

		**Demographics**					
		**Cx-**	**Cx+**				
		**(n = 21)**	**Sm- (n = 25)**	**Sm+ (n = 53)**	***P*** ^***a***^	**All Cx+ (n = 78)**	***P*** ^***b***^
**Age, years, median [25%ile, 75%ile]**		37 [31, 51]	38 [27, 50]	39 [31, 53]	0.31	39 [29, 52]	0.71
**Male sex (%)**		18 (86)	13 (52)*	38 (72)	0.04	51 (65)	0.07
**Race** ^**c**^ **(%)**					0.29		0.30
	**Asian**	10 (53)	10 (42)	22 (42)		32 (42)	
	**Black**	1 (5)	3 (13)	15 (29)		18 (24)	
	**Caucasian**	1 (5)	2 (8)	3 (6)		5 (7)	
	**Hispanic**	7 (37)	9 (38)	12 (23)		21 (28)	
**Foreign-born (%)**		16 (76)	18 (75)	43 (83)	0.67	61 (80)	0.76
**Years in US** ^**d**^ **, median [25%ile, 75%ile]**		2 [1, 7]	5 [3, 5]	6 [2, 16]	0.22	5 [2, 12.5]	0.15
		**Symptoms**					
		**Cx-**	**Cx+**				
		**(n = 20)**	**Sm- (n = 23)**	**Sm+ (n = 46)**	***P*** ^***a***^	**All Cx+ (n = 69)**	***P*** ^***b***^
**Cough (%)**		14 (70)	20 (87)	43 (94)*	< 0.05	63 (91)	0.02
**Sputum Production (%)**		6 (30)	12 (52)	32 (70)**	0.01	44 (64)	<0.01
**Fever (%)**		8 (40)	11 (48)	27 (59)	0.34	38 (55)	0.24
**Night Sweats (%)**		8 (40)	7 (30)	28 (61)	0.04	35 (51)	0.40
**Weight Loss (%)**		5 (25)	10 (44)	27 (59)*	0.04	37 (54)	0.02
**Hemoptysis (%)**		4 (20)	6 (26)	13 (28)	0.78	19 (28)	0.50
**Any Symptom (%)**		16 (80)	22 (96)	45 (98)*	0.03	67 (97)	0.02

Culture and smear results were based off of the first three sputum samples. Symptoms were self-reported at the time of initial presentation. Ten of the 99 subjects had missing data on symptoms, 1 was Cx- and 9 were Cx+. Abbreviations: Cx-, culture-negative; Cx+, culture-positive; Sm-, smear-negative; Sm+, smear-positive.

a Determined for 3-group comparison: Cx- vs. Sm-Cx+ vs. Sm+Cx+.

b Determined for 2-group comparison: Cx- vs. All Cx+.

c Race information was available for 95/99 subjects.

d Among foreign-born only. Years in US information was available for 63/99 subjects.

* *P* < 0.05 for *a priori* comparison of group to Cx-.

** *P* < 0.01 for *a priori* comparison of group to Cx-.

### Radiology

All 99 subjects had chest X-ray data; additionally, 57 subjects had chest CT data. No significant differences in abnormal chest X-ray findings were observed between Cx- and Cx+ subjects. However, Cx- subjects were significantly more likely to have a chest CT evaluation compared to Cx+ subjects (Table 3). Cavitation on chest CT occurred significantly less frequently in Cx- subjects compared with Cx+ (Table 3). In the three-group comparison, there was further a significant difference in the frequency of cavitation, with Cx- having a significantly lower frequency compared to both Sm- Cx+ subjects and Sm+ Cx+ subjects (Table 3). This trend for increasing observation of cavitation was significant in both X-ray (*P* for trend = 0.04) and CT (*P* for trend < 0.01). The dataset is included as a supplementary file (S1 Dataset).

**Table 3:** Radiological Findings in Patients with Pulmonary Tuberculosis.

	**Chest X-Ray**					
	**Cx-**	**Cx+**				
	**(n = 21)**	**Sm- (n = 25)**	**Sm+ (n = 53)**	***P*** ^***a***^	**All Cx+ (n = 78)**	***P*** ^***b***^
**Upper Lobe Inf (%)**	15 (71)	15 (60)	39 (74)	0.47	54 (69)	>0.99
**Unilateral Inf (%)**	10 (48)	11 (44)	22 (42)	0.89	33 (42)	0.66
**Multilobar Inf (%)**	6 (29)	10 (40)	28 (53)	0.15	38 (49)	0.10
**Nodular Inf (%)**	13 (62)	15 (60)	25 (47)	0.39	40 (51)	0.39
**Miliary Inf (%)**	1 (5)	1 (4)	2 (4)	>0.99	3 (4)	>0.99
**Cavitation (%)**	2 (10)	4 (16)	16 (30)	0.13	20 (26)	0.15
**Chest CT scan received (%)**	17 (81)	17 (68)	23 (43)**	0.01	40 (51)	0.02
	**Chest CT**					
	**Cx-**	**Cx+**				
	**(n = 17)**	**Sm- (n = 17)**	**Sm+ (n = 23)**	***P*** ^***a***^	**All Cx+ (n = 40)**	***P*** ^***b***^
**Upper Lobe Inf (%)**	13 (77)	14 (82)	18 (78)	>0.99	32 (80)	0.74
**Unilateral Inf (%)**	8 (47)	4 (24)	6 (26)	0.26	10 (25)	0.10
**Multilobar Inf (%)**	8 (47)	12 (71)	14 (61)	0.37	26 (65)	0.21
**Nodular Inf (%)**	13 (77)	14 (82)	17 (74)	0.92	31 (78)	>0.99
**Miliary Inf (%)**	3 (18)	3 (18)	4 (17)	>0.99	7 (18)	>0.99
**Cavitation (%)**	2 (12)	10 (59)**	17 (74)***	<0.01	27 (68)	<0.01

Culture and smear results were based off of the first three sputum samples. Radiological findings were based on the first chest X-ray obtained at initial presentation, and if performed, on the initial chest CT scan. Abbreviations: CT, computed tomography; Cx-, culture-negative; Cx+, culture-positive; Sm-, smear-negative; Sm+, smear-positive; Inf, infiltrates.

a Determined for 3-group comparison: Cx- vs. Sm-Cx+ vs. Sm+Cx+.

b Determined for 2-group comparison: Cx- vs. All Cx+.

** *P* < 0.01 for *a priori* comparison of group to Cx-.

*** *P <* 0.001 for *a priori* comparison of group to Cx-.

## Discussion

Our study elucidates the clinical and radiographic presentation of Cx- PTB. Consistent with our hypothesis, our data show that patients with Cx- PTB present with fewer symptoms and less extensive radiographic abnormalities compared to those with Cx+ PTB—specifically, less cough, sputum production, weight loss, and cavitary lesions. These findings emphasize that medical providers should neither rely on Mtb culture as the gold standard tests for TB nor expect a clinical presentation with the symptoms characteristic of advanced PTB in order to diagnose TB and initiate treatment at a very early state. Such awareness is critical, as it is possible to treat Cx- PTB, defined as the initial three sputum cultures negative for Mtb, with fewer drugs and shorter duration [5, 6], thereby facilitating patience adherence, diminishing drug adverse effects, and reducing transmission.

Our study followed the CDC guidelines and used the first three sputum samples to classify the positivity of the smears and cultures [2, 5], in contrast to the two sputum samples recommended by the WHO [13, 14]. This makes our Cx- definition clinically pertinent in a wide array of settings as most medical care providers order no more than three sputum smears and cultures for TB suspects. The use of three induced concentrated sputum samples further increases sensitivity for detection of Mtb, thereby supporting that our subjects truly had paucibacillary disease. Such paucibacillary Cx- PTB may be more recognized in settings with sufficient resources for extensive work-up. For example, we observed that CT scans were significantly more frequently performed in Cx- compared to Cx+ PTB subjects likely because chest CT scans can reveal abnormalities not seen on chest X-rays and can thus aid in diagnosis of Cx- PTB. Additionally, availability of other diagnostic tools further allowed us to exhaustively exclude other respiratory diseases. These factors allowed for the detection and characterization of a Cx- disease state, which we believe should be characterized as an early disease state since it plausibly precedes Cx+ TB.

The reduced frequency of symptoms and cavitation that we observed in Cx- compared with Cx+ PTB is consistent with the observed gradient of TB infection and disease spectrum described by us and others [8, 15, 16]. Contrary to the Hong Kong based study in the early 1980s [7], we did not find significant differences in hemoptysis. On the other hand, consistent with their data, we also found a lower frequency of cavitation in Cx- PTB compared with Cx+ subjects [7]. These findings further support that, in HIV-uninfected individuals, Cx- PTB is an early disease state with low mycobacterial burden. It is also conceivable that Cx- and Cx+ TB patients could differ in their immune response to TB, but studies investigating this area are lacking.

Recognizing and diagnosing Cx- TB is clinically challenging. Although cough, sputum production, and cavitation are the hallmark manifestations of PTB [2, 17, 18], our study highlights that they might not be present in Cx- PTB. In resource-limited settings, even if respiratory symptoms are present, patients without bacteriologic evidence for TB (commonly restricted to sputum microscopy) are typically treated empirically for other bacterial infections without further follow-up unless they return due to unresolved or progressive symptoms. Consequently, early states of TB are often neither suspected nor empirically treated in many settings, especially if cough and sputum production are absent. In a review of deaths due to respiratory infections in Africa, Bates et al. found that premortem clinical diagnosis of TB in both HIV-infected and -uninfected patients has a limited sensitivity of 67% when using postmortem pathological diagnosis as the gold standard [19]. Further autopsy studies among populations with little-to-no HIV-coinfection have shown even higher proportions (~50%) of undiagnosed TB due low clinical suspicion premortem [20–25]. These missed TB diagnoses were likely due to early and less symptomatic states of TB premortem, consistent with our study, highlighting the need for increased awareness of the paucity of symptoms and radiographic abnormalities of early TB states.

The findings of our study suggest that additional diagnostic tools facilitating the recognition of Cx- TB and other early TB states are needed. Depending on context, more than three sputum samples and investigation of samples other than sputum, as well as chest CT, bronchoscopy, and biopsies should be considered for further work-up in subjects in whom TB is highly suspected. However, resources for such work-up are limited in many TB endemic settings. In their Global Tuberculosis Report 2014, the WHO declares that “one of the most urgently needed tests is a rapid biomarker-based test that can diagnose PTB and ideally also extrapulmonary TB using non-sputum samples” [1]. Ideally, such TB biomarkers should also be able to detect the earliest and most difficult to diagnose TB cases, and should be suitable for use in resource-limited settings. This need has been recognized by some investigators such as Kaforou et al. who recently noted that one of their TB biomarker study’s limitations was the restriction to using only Cx+ TB [26].

As there is no gold standard test for the diagnosis of Cx- PTB available, our study was limited by the lack of absolute certainty that all of our Cx- subjects who improved on antituberculous treatment definitely had PTB. Nevertheless, in agreement with the CDC [5], we are confident that the subjects in whom TB diagnosis was solely based on clinical and radiologic improvement on antituberculous treatment, the combination of such with TB risk factors, key radiological abnormalities, and exclusion of other respiratory diseases, indicated that those subjects had Cx- TB. Another limitation of our study was the lack of sufficient power to detect significant differences in characteristics such as symptom duration due to our modest sample size.

### Conclusions

Our characterization of Cx- PTB should raise awareness that neither culture, an imperfectly sensitive gold standard, nor the hallmark symptoms for TB are invariably associated with clinically important disease. This early form of TB is likely to be grossly underestimated and untreated worldwide, highlighting the need for biomarkers that can detect very early states of the disease. Larger studies are needed to validate our findings.

## Supporting Information

S1 Dataset Culture-Negative Pulmonary Tuberculosis Dataset. (CSV)
